# Socioeconomic inequalities in primary-care and specialist physician visits: a systematic review

**DOI:** 10.1186/s12939-020-01375-1

**Published:** 2021-02-10

**Authors:** Sara Lena Lueckmann, Jens Hoebel, Julia Roick, Jenny Markert, Jacob Spallek, Olaf von dem Knesebeck, Matthias Richter

**Affiliations:** 1 Institute of Medical Sociology, Medical Faculty, Martin Luther University Halle-Wittenberg, Magdeburger Str. 8, 06112 Halle (Saale), Germany; 2 University Hospital Halle (Saale), Ernst-Grube-Str. 40, 06120 Halle (Saale), Germany; 3 Division of Social Determinants of Health, Department of Epidemiology and Health Monitoring, Robert Koch Institute, Berlin, Germany; 4 Department of Public Health, Brandenburg University of Technology Cottbus-Senftenberg, Senftenberg, Germany; 5 Institute of Medical Sociology, University Medical Center Hamburg-Eppendorf, Hamburg, Germany

**Keywords:** Social inequalities, Socioeconomic Status, Primary health care, Access to health care

## Abstract

**Background:**

Utilization of primary-care and specialist physicians seems to be associated differently with socioeconomic status (SES). This review aims to summarize and compare the evidence on socioeconomic inequalities in consulting primary-care or specialist physicians in the general adult population in high-income countries.

**Methods:**

We carried out a systematic search across the most relevant databases (Web of Science, Medline) and included all studies, published since 2004, reporting associations between SES and utilization of primary-care and/or specialist physicians. In total, 57 studies fulfilled the eligibility criteria.

**Results:**

Many studies found socioeconomic inequalities in physician utilization, but inequalities were more pronounced in visiting specialists than primary-care physicians. The results of the studies varied strongly according to the operationalization of utilization, namely whether a physician was visited (probability) or how often a physician was visited (frequency). For probabilities of visiting primary-care physicians predominantly no association with SES was found, but frequencies of visits were higher in the most disadvantaged. The most disadvantaged often had lower probabilities of visiting specialists, but in many studies no link was found between the number of visits and SES.

**Conclusion:**

This systematic review emphasizes that inequalities to the detriment of the most deprived is primarily a problem in the probability of visiting specialist physicians. Healthcare policy should focus first off on effective access to specialist physicians in order to tackle inequalities in healthcare.

**PROSPERO registration number:**

CRD42019123222.

**Supplementary Information:**

The online version contains supplementary material available at 10.1186/s12939-020-01375-1.

## Background

Health inequalities, precisely inverse associations between socioeconomic status (SES) and morbidity and mortality, are well analysed and described [1]. Further, numerous studies prove evidence for vertical inequalities in utilization of healthcare according to education, income and occupation, which represent SES. In order to shed more light on the role of healthcare in explaining health inequalities, it is crucial to examine socio-economic inequalities in the utilization of treatment in a more differentiated way. It has been shown that socioeconomic inequalities in healthcare are present in both universal and non-universal healthcare system, and existence does not depend on the type and financing of health systems [2–4]. Distinctions were more likely to be found according to the different dimensions of healthcare for which inequalities are analysed. So far, international evidence on socioeconomic inequalities in treatment was mainly summarized on disease-specific, or country-specific basis and indicate that lower SES is associated with poor diabetes management, lower achievement of glycaemic control targets, and reduced visits of diabetes clinics and ambulatory care facilities for treatment of diabetes [3, 5]. In cancer patients, lower SES is associated with receiving less often (neo) adjuvant therapy for colorectal cancer, [6] and with receiving less often any treatment, surgery and chemotherapy for lung cancer [2]. In coronary heart disease patients SES was often associated with lower access to coronary procedures [4]. Nevertheless, SES was only partly associated with receiving radiotherapy and chemotherapy in colorectal cancer patients, [6] or with access to drug treatment and cardiac rehabilitation in coronary heart disease patients, [4] and not associated with radiotherapy for lung cancer [2]. In contrast, in diabetes patients, it was found that lower SES was associated with more visits to a diabetologist, and more often GP consultations [3]. For Germany it was summarized that higher status groups presented higher utilization in terms of specialist consultations and prevention services [7]. It should be noted, however, that systematic reviews often summarize studies that use different operationalisations of SES and healthcare utilization, and may therefore be difficult to compare.

In order to tackle inequalities in utilization of healthcare, we need to gain a better understanding of healthcare inequalities. More detailed evidence is needed in which domains of healthcare and indicators of utilisation and SES are specified [8]. Despite disease- and country-specific systematic reviews on socioeconomic inequalities, only two reviews summarized the international evidence of inequalities in utilization rates in the general population [8, 9]. However, the first limited their analyses to home health services in developed countries, and found that utilization of home health services in the general population was notably lower for persons with high compared to low SES [8]. The second limited the analyses to healthcare utilization rates in the elderly population and found that the association with SES varied strongly according to the type of healthcare analysed. While elderly patients with low SES were advantaged in home visits, they were disadvantaged in dental and medical appointments, and no association with SES was found for hospitalization rates and emergency use [9].

So far, it is evident that socioeconomic inequalities in utilization rates differ depending on the domain of healthcare analysed. Nevertheless, the evidence on socioeconomic differences in physician utilization in the general adult population has not been summarized so far. Moreover, individual studies suggest that inequalities in physician visits differ depending on whether utilization of primary-care or specialist physicians is analysed [10–12]. Therefore, enhanced knowledge is needed (1) if socioeconomic inequalities in visiting primary-care or specialist physicians do exist; and (2) if divergent results of inequalities in physician visits can be explored depending on methodical diversity, e.g., operationalization of SES or utilization.

## Methods

This review aims to summarize the evidence on socioeconomic inequalities in consulting primary-care and specialist physicians in the general adult population in high-income countries. Studies from low- and middle income countries (defined by The World Bank 2019 [13]) were excluded as the nature of and issues related to healthcare utilization and health-care system differ significantly from health care systems in high-income countries. To perform this review, we searched the electronic databases Medline and Web of Science to identify relevant studies. In addition, we manually searched the reference lists of all included articles for further potentially relevant studies. The search was conducted in January 2019 and limited to articles published in either English or German within the last 15 years. Different combinations of keywords related to (a) primary-care or specialist physicians, (b) inequalities, and (c) SES were used for the search (see additional file 1).

### Study selection and eligibility criteria

The identified records were independently screened by two researchers for eligibility criteria in three consecutive steps: titles, abstracts, full texts. SLL conducted the screening at any step, JH conducted the title- and abstract screening, and JR and JM each conducted half of the full text screening. After each step a joint decision was reached through discussions in cases of disagreement. The criteria used to identify articles of interest limited the search results to original quantitative studies. An article was included in the review if it met the following criteria: (a) analysing the general population aged 15 years or older in a high-income country; (b) analysing any SES indicator (income, education, occupation, social class, or any combination of these indicators) based on individual data; (c) analysing utilization of primary-care, or specialist physicians, or both independently from each other (d) presenting quantitative original data on differences in utilization between at least two different SES groups. The following exclusion criteria were applied: (a) specific populations, namely disease- or SES-specific; (b) differences in race, rurality, insurance status, financial barriers, or employment status; (c) utilization of medical interventions, dentists, inpatient treatment, healthcare in general, or of physicians without differentiating between primary and specialized care; (d) SES or utilization based on area data, or not linked to the individual; (e) conference abstracts and comments.

### Data extraction and quality assessment

Data extraction was conducted by SLL, and checked by JR or JM. The following information was extracted from texts, tables, and figures of the included studies: author, year, countries, database, number of participants, participant’s age, physician (primary care or specialist), measurement of utilization, measurement of SES, confounder variables, and the result if an association of SES with physician visits has been found. As the included studies analysed different aspects, it was difficult to compare them in a common scheme that would account for all differences. Consequently, assumptions and simplifications had to be made in order to compare the studies. The results in the tables were abstracted to the most relevant finding analysing if a relationship (and the direction) between SES and utilization of primary-care or specialist physicians was found with the following simplifications:

1. the results comparing the highest SES with the lowest SES (when more than two SES-groups were compared);
2. significant differences at a *p* ≤ 0.05 or lower (when several *p* values were designated);
3. the most recent findings (from studies analysing trends of socioeconomic inequalities);
4. the results from the best fitting final model (if an analysis was conducted using different types of adjustments);
5. the results including the broadest variety of the population (if subgroups, e.g. private and public healthcare, were analysed)

Further, to simplify the description of the extracted information and the comparison:

1. we only report the results from high-income countries and report only the most frequent result (if several analyses have been conducted for more than four countries);
2. we dichotomized adjustment variables to “↑” if adjustments were made for at least gender, age and any general health variable; and “↓” if the required need adjustments were not made, including only adjusting for age, gender, and mental health.

Risk of bias was assessed in accordance with RoBANS, [14] and assessed independently by (1) SLL and (2) either JR or JM. The assessments were subsequently discussed to achieve a consensus regarding the rating of each domain in each included article. In a joint decision it was defined that register and national survey data are defined to present “low risk of bias” for the selection of participants, but “high risk” when only sub-populations were analysed without rationale. Second, confounding variables presenting a “low risk of bias” are age, gender, and a minimum of one need-variable of chronic diseases or self-rated health. Further, register data and standardized questionnaires measuring self-reported values are defined to be a “low risk for bias” for measurement of exposure. Fourth, register and national survey data are defined to present “low risk of bias” for the blinding of outcome measure. Fifths, the risk of bias for incomplete outcome data was defined unclear, when missing values were not mentioned or imputed, but high risk when missing values were evident but not tested, and defined low risk when missing values were mentioned and tested. Lastly, for secondary data analyses and analyses of register, panel or national survey data without a study protocol, selective outcome reporting was rated “low risk of bias” when descriptions in the methods section match with the results section.

## Results

**Fig. 1:**
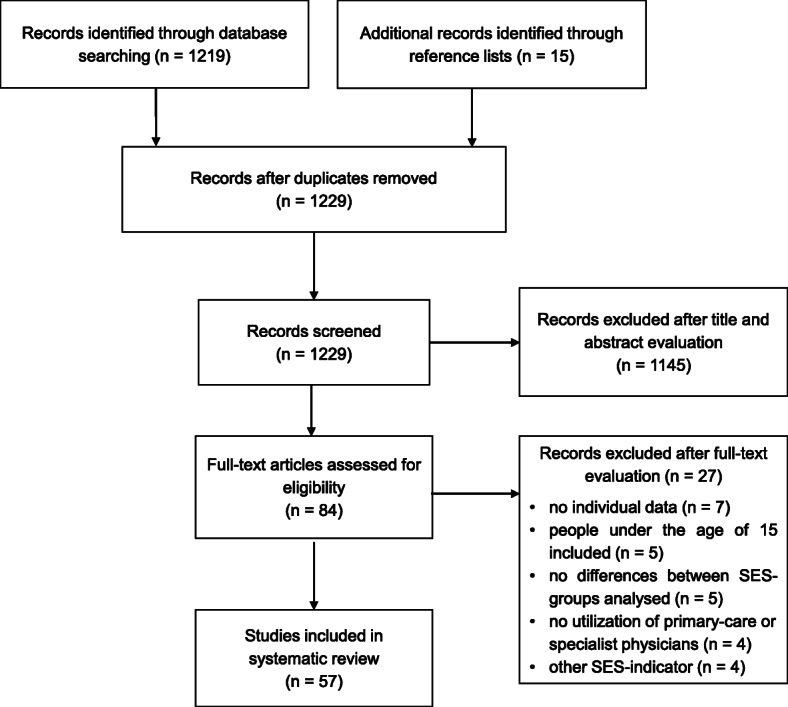
Flowchart of the systematic literature research

We found 1229 unique abstracts published between January 2004 and December 2018. Among these, 57 examined socioeconomic differences in physician visits and met all inclusion criteria. The flowchart of the study selection procedure is presented in Fig. 1. Most studies were based on register-data or secondary data from population surveys (see Table 1). In total, the studies comprised data from 32 high-income countries, of which seven were non-European countries, namely, Australia, Canada, Chile, Hong Kong, Israel, New Zealand, and the USA. Whereas three studies analysed pooled data from several European countries, the majority analysed data from one country (*n*=44), or several countries separately (*n*=10). These 54 studies most often reported data from Spain (*n*=15), Germany (*n*=14), and Belgium (n=10).

**Table 1:** Characteristics of the 57 studies included in the systematic review

Author	Year	Countries	database (target population)	number of participants	age of participants
Abasolo, Saez, López-Casasnovas [15]	2017	Spain	Spanish National Health Survey 2011/12	19,935	≥ 15 years
Agerholm et al. [16]	2013	Sweden	Public Health Survey in Stockholm County 2006, Stockholm County Council’s administrative database 2007, Longitudinal integration database for health insurance and labor market studies	31,848	25 to 84 years
Allin [17]	2008	Canada	Canadian Community Health Survey 2003	104,510	≥ 15 years
Asada, Kephart [18]	2007	Canada	Canadian Community Health Survey 2000/1	133,300	≥ 20 years
Bago d’Uva, Jones, van Doorslaer [19]	2009	Austria, Belgium, Denmark, Finland, Greece, Ireland, Italy, Netherlands, Portugal, Spain	European Community Household Panel User Database 1994–2001	N.A.	≥ 16 years
Baron-Epel, Garty, Green [20]	2007	Israel	Israel National Health Survey 2003/04	9512	≥ 21 years
Beckman, Anell [21]	2013	Sweden	Skåne Regional Council and Statistics Sweden 2010/11, Statistics Sweden 2009	828,988	25 to 84 years
Bergmann, Kalcklösch, Tiemann [22]	2005	Germany	Telephone Health Survey 2003	8318	≥ 18 years
Bourke [23]	2009	Ireland	Living in Ireland survey 2001	6518	≥ 16 years
Bremer, Wübker [24]	2013	Germany	Survey of Health, Aging and Retirement in Europe 2004–2006	2861	≥ 50 years
Bremer, et al. [25]	2018	pooled data from 16 European countries	Survey of Health, Aging and Retirement in Europe 2010/11	56,989	≥ 50 years
Crespo-Cebada, Urbanos-Garrido [26]	2012	Spain	Survey of Health, Aging and Retirement in Europe 2006/07	1860	≥ 50 years
Devaux, de Looper [27]	2012	Austria, Belgium, Canada, Czech Republic, Denmark, Estonia, Finland, France, Hungary, Ireland, New Zealand, Poland, Slovak Republic, Slovenia, Spain, Switzerland, UK	European Health Interview Surveys (2006/07, 2007, 2008 or 2009) other national health interview surveys (2005, 2006/07, 2007, 2007/08, 2008 or 2009)	N.A.	≥ 15 years
Fjaer, et al. [28]	2017	Austria, Belgium, Czech Republic, Denmark, Estonia, Finland, France, Germany, Hungary, Ireland, Israel, Lithuania, Netherlands, Norway, Poland, Portugal, Slovenia, Spain, Sweden, Switzerland, UK	European social survey 2014	31,971	25 to 75 years
Garrido-Cumbrera, et al. [29]	2010	Spain	Spanish National Health Survey 2006	29,478	≥ 16 years
Glazier et al. [30]	2009	Canada	Canadian Community Health Survey 2000/01, Physician claim files in 2002/03 and 2003/04	25,558	20 to 79 years
Gonzalez-Alvarez, Barranquero [31]	2009	Spain	European Community Household Panel 1994–2001	15,076	≥ 16 years
Grasdal, Monstad [32]	2011	Norway	Survey of Living Conditions 2005, Administrative records 2005	3002	16 to 69 years
Gruber, Kiesel [33]	2010	Germany	Survey of Health, Ageing and Retirement in Europe 2004	2260	50 to 90 years
Habicht, Kunst [34]	2005	Estonia	Survey of Living Conditions 1999	3990	25 to 74 years
Hansen, et al. [35]	2012	Norway	Tromsø Study 2007/08	12,982	30 to 87 years
Hoebel, et al. [12]	2016	Germany	German Health Interview and Examination Survey for Adults 2008–2011	6754	18 to 69 years
Hoeck, et al. [36]	2011	Belgium	Belgian Health Interview Survey 2001–2004	4494	≥ 65 years
Hoeck, et al. [37]	2013	Belgium	Belgian Health Interview Survey 2001–2004	19,563	≥ 16 years
Korda, et al. [38]	2009	Australia	Australian Longitudinal Study of Women’s Health 2004	10,905	53 to 58 years
La Parra-Casado, et al. [39]	2018	Spain	Spanish National Health Survey 2011/12	21,650	≥ 16 years
Lichte [40]	2017	Germany	random sample survey of general practitioner attenders 2015/16	519	≥ 18 years
Lostao, et al. [41]	2011	UK, Spain	General Household Survey 2004/05 Spanish National Health Survey 2003	36,488	≥ 16 years
Lu, et al. [42]	2007	Hong Kong	Thematic Household Survey 2002	19,522	≥ 16 years
Masseria, Giannoni [43]	2010	Italy	Multiscopo Survey 1999/2000	109,964	> 16 years
McDonald, Conde [44]	2010	Canada	Canadian Community Health Survey 2002/03	39,974	55 to 79 years
Mosquera, et al. [45]	2017	Sweden	Health on Equal Terms survey 2014	3016	16 to 25 years
Nolan [46]	2007	Ireland	Living in Ireland Survey 1995–2001	49,237	≥ 16 years
Palència, et al. [47]	2013	Spain	Spanish National Health Survey 2006	20,478	≥ 16 years
Põlluste, Kalda, Lember [48]	2009	Estonia	random sample survey of general population 2005	182	65 to 74 years
Rattay et al. [49]	2013	Germany	German Health Interview and Examination Survey for Adults 2008–2011	8152	18 to 79 years
Regidor, et al. [50]	2008	Spain	Spanish National Health Survey 2003/04	18,837	16 to 74 years
Reibling, Wendt [51]	2010	Austria, Belgium, Denmark, France, Germany, Greece, Italy, Netherlands, Spain, Sweden, Switzerland	Survey of Health, Ageing and Retirement in Europe 2004	26,808	≥ 50 years
Rogowski et al. [52]	2008	USA	random sample survey of Medicare enrollees 2000; administrative data	4600	≥ 65 years
Ryvicker, Gallo, Fahs [53]	2012	USA	random sample survey of community-dwelling older senior center attendees 2008	1870	60 to 99 years
San Sebastian, Mosquera, Gustafsson [54]	2017	Sweden	Health on equal terms survey 2014 Statistics Sweden	24,889	19 to 84 years
Schnitzer, et al. [55]	2011	Germany	Representative sample survey of the population with statutory health insurance 2010	5232	18 to 79 years
Schulz [56]	2016	pooled data from 13 European countries	Survey of Health, Aging, and Retirement 2004/05–2006/07	48,065	≥ 40 years
Stirbu, et al. [11]	2011	Belgium, Estonia, France, Germany, Hungary, Ireland, Latvia, Netherlands, Norway	several national health surveys between 1995 and 2004	104,503	≥ 15 years
Suominen-Taipale, et al. [57]	2004	Finland, Norway	The Health Study of Nord-Trondelag, HUNT 1995–1997 FINRISK-97 senior survey 1997	9202	65 to 74 years
Tavares, Zantomio [58]	2017	Italy, Spain, Portugal	Survey of Health, Aging and Retirement in Europe 2011	9049	≥ 50 years
Terraneo [10]	2015	pooled data from 12 European countries	Survey of Health, Aging and Retirement in Europe 2007–2009	16,431	≥ 50 years
Thode et al. [59]	2005	Germany	German Health Interview and Examination Survey for Adults 1998	7124	18 to 79 years
Tille, et al. [60]	2017	Germany	random sample survey of the general population 2006–2016	42,925	≥ 18 years
van Doorslaer, Koolman, Jones [61]	2004	Austria, Belgium, Denmark, Germany, Greece, Ireland, Italy, Luxemburg, Netherlands, Portugal, Spain, UK	European community household panel 1996	N.A.	≥ 16 years
van Doorslaer, Masseria, Koolman [62]	2006	Austria, Belgium, Canada, Denmark, Finland, France, Germany, Greece, Hungary, Ireland, Italy, Netherlands, Norway, Portugal, Spain, Switzerland, UK	European community household panel and other nationally representative surveys 1996–2002	N.A.	≥ 16 years
van Ourti [63]	2004	Belgium	panel study of Belgian households 2001	4809	> 15 years
Vasquez, Paraje, Estay [64]	2013	Chile	national socio-economic characterization survey 2009	246,924	≥ 18 years
Vedsted et al. [65]	2004	Denmark	intervention study of general practitioner attenders	2526	20 to 64 years
Vedsted, Olesen [66]	2005	Denmark	intervention study of general practitioner attenders	2211	20 to 64 years
Vikum, et al. [67]	2013	Norway	Nord-Trøndelag Health Study 2006/08 register data	46,860	≥ 20 years
Vikum, Krokstad, Westin [68]	2012	Norway	Nord-Trøndelag Health Study 2006/08 register data	44,755	≥ 20 years

Overall, 70% (*n*=40) of the studies analysed both primary-care and specialist physician visits, another 25% (n=14) of the studies only primary-care physician visits, and 5% (*n*=3) only specialist physician visits (Table 2). The definition of primary- and specialist care differed between the studies and health-care systems. Primary-care implied family physicians, and/or general practitioners, but in some cases after excluding prevention services, child or maternity care, physicians at healthcare centres, or internal medicine physicians. Specialist care was defined as medical outpatient specialists, any specialist except while being hospitalized, or generally physician visits at the hospital without being hospitalized. Utilization of physicians was measured according to probability (having visited a physician or not) in 72% (*n*=41) of the studies, according to frequency (number of visits) in 37% (*n*=21) of the studies, or according to conditional frequency (number of visits conditional to having visited a physician at least once) in 30% (*n*=17) of the studies. 95% (*n*=54) of the studies adjusted the analysis for need according to at least gender, age, and either self-rated health or chronic conditions. SES was measured by income (58%; *n*=33) and/or education (54%; *n*=31) in most of the studies. 4% (*n*=2) of the studies measured SES by income, education, and occupation; 9% (n=5) of the studies only by occupation; and 7% (*n*=4) of the studies by an SES-index. The period for which physician utilization was reported by the participants, ranged from two weeks to two years. 68% (*n*=39) of the studies analysed utilization rates within the last 12 months, 14% (*n*=8) of the studies within the last four weeks or one month, 11% (*n*=6) of the studies within the last three months, 5% of the studies within the last two months, each 4% (*n*=2) of the studies within the last two weeks, and last two years, and 2% (*n*=1) of the studies within the last six months. Most of the 57 studies have carried out several calculations (for different countries, age groups, utilization or SES measures; see Table 2). Therefore, and through rounding the percentages are more than 100%. The following results are based on a total of 548 different analyses.

**Table 2:** Results on relationships between socioeconomic status and utilization of primary-care and specialist physicians

author	countries	age group	time	physician	utilisation	SES measure	need-adjusted	result
Abasolo, Saez, López-Casasnovas [15]	Spain	≥ 15 years	4 weeks	primary care	frequency	household income	↓	o
				specialist				o
Agerholm et al. [16]	Sweden	25 to 64 years	12 months	primary care	frequency	adjusted household income	↑	o^4^
				specialist				+
		65 to 84 years		primary care				o^4^
				specialist				+
Allin [17]	Canada	≥ 15 years	12 months	primary care	probability	adjusted household income	↑	+
					frequency			o
				specialist	probability			+
					frequency			+
Asada, Kephart [18]	Canada	≥ 20 years	12 months	primary care	probability	education	↑	+
						adjusted household income		+
					conditional frequency	education		–
						adjusted household income		–
				specialist	probability	education		+
						adjusted household income		+
					conditional frequency	education		+
						adjusted household income		o
Bago d’Uva, Jones, van Doorslaer [19]	Austria, Belgium, Denmark, Finland, Greece, Ireland, Italy, Netherlands, Portugal, Spain	≥ 16 years	12 months	primary care	frequency	adjusted household income	↑	−^5^
				specialist				+
Baron-Epel, Garty, Green [20]	Israel	≥ 21 years	4 weeks	primary care	probability	education	↑	–
						household income		–
				specialist		education		+
						household income		o
Beckman, Anell [21]	Sweden	25 to 44 years	2 years	primary care	probability	household income	↓	+
		45 to 64 years						o^4^
		65 to 84 years						+
Bergmann, Kalcklösch, Tiemann [22]	Germany	≥ 18 years	12 months	primary care	frequency	index	↑	–
Bourke [23]	Ireland	≥ 16 years	12 months	primary care	probability	adjusted household income	↑	o
					frequency			–
					conditional frequency			–
				specialist	probability			+
					frequency			o
					conditional frequency			–
Bremer, Wübker [24]	Germany	≥ 50 years	12 months	primary care	probability	education	↑	o
						adjusted household income		o
					conditional frequency	education		o
						adjusted household income		o
				specialist	probability	education		o
						adjusted household income		+
					conditional frequency	education		o
						adjusted household income		+
Bremer, et al. [25]	Pooled Data from 16 European Countries	≥ 50 years	12 months	primary care	frequency	education	↑	–
Crespo-Cebada, Urbanos-Garrido [26]	Spain	≥ 50 years	12 months	primary care	probability	education	↑	o
						adjusted household income		o
					conditional frequency	education		o
						adjusted household income		o
Devaux, de Looper [27]	Austria, Belgium, Canada, Czech Republic, Denmark, Estonia, Finland, France, Hungary, Ireland, New Zealand, Poland, Slovak Republic, Slovenia, Spain, Switzerland, UK	≥ 15 years	12 months^1^	primary care	probability	adjusted household income	↑	o^5^
					frequency			o^5^
				specialist	probability			+ ^5^
					frequency			+ ^5^
Fjaer, et al. [28]	Austria, Belgium, Czech Republic, Denmark, Estonia, Finland, France, Germany, Hungary, Ireland, Israel, Lithuania, Netherlands, Norway, Poland, Portugal, Slovenia, Spain, Sweden, Switzerland, UK	25 to 75 years	12 months	primary care	probability	education	↑	o^5^
				specialist				+ ^5^
Garrido-Cumbrera, et al. [29]	Spain	≥ 16 years	4 weeks	primary care	probability	occupation	↑	–
				specialist				+
Glazier et al. [30]	Canada	20 to 79 years	2 years	primary care	probability	education	↑	o
						adjusted household income		o
					conditional frequency	education		–
						adjusted household income		–
				specialist	probability	education		+
						adjusted household income		o
					conditional frequency	education		+
						adjusted household income		o
Gonzalez-Alvarez, Barranquero [31]	Spain	≥ 16 years	12 months	primary care	probability	education	↑	–
						adjusted household income		o
					frequency	education		–
						adjusted household income		o
					conditional frequency	education		–
						adjusted household income		o
				specialist	probability	education		+
						adjusted household income		+
					frequency	education		+
						adjusted household income		+
					conditional frequency	education		o
						adjusted household income		o
Grasdal, Monstad [32]	Norway	16 to 69 years	12 months	primary care	probability	adjusted household income	↑	o
					conditional frequency			o
				specialist	probability			+ ^6^
					conditional frequency			o
Gruber, Kiesel [33]	Germany	50 to 90 years	12 months	specialist	probability	education	↑	o^4^
						adjusted household income		o^4^
					frequency	education		o
						adjusted household income		o
Habicht, Kunst [34]	Estonia	25 to 74 years	6 months	primary care	probability	education	↑	o
						adjusted household income		+
				specialist		education		+
						adjusted household income		+
Hansen, et al. [35]	Norway	30 to 87 years	12 months	primary care	probability	education	↑	o
						household income		o
						occupation		o
					conditional frequency	education		o^4^
						household income		–
						occupation		o
Hoebel, et al. [12]	Germany	18 to 69 years	12 months	primary care	probability	index	↑	o
					conditional frequency			–
				specialist	probability			+
					conditional frequency			o
Hoeck, et al. [36]	Belgium	≥ 65 years	2 months	primary care	probability	education	↑	o
						adjusted household income		o
				specialist		education		o
						adjusted household income		o
Hoeck, et al. [37]	Belgium	≥ 16 years	2 months	primary care	probability	education	↑	o
						adjusted household income		o
					conditional frequency	education		o
						adjusted household income		o
				specialist	probability	education		+
						adjusted household income		o
					conditional frequency	education		o
						adjusted household income		o
		≥ 65 years		primary care	probability	education	↑	o
						adjusted household income		o
					conditional frequency	education		o
						adjusted household income		o
				specialist	probability	education		+
						adjusted household income		o
					conditional frequency	education		o
						adjusted household income		o
Korda, et al. [38]	Australia	53 to 58 years	12 months	primary care	probability	occupation	↑	o
					conditional frequency			o
				specialist	probability			+
					conditional frequency			o
La Parra-Casado, et al. [39]	Spain	≥ 16 years	4 weeks	primary care	probability	occupation	↑	o
Lichte [40]	Germany	≥ 18 years	3 months	primary care	conditional frequency	education	↑	o
						household income		o
Lostao, et al. [41]	UK	≥ 16 years	2 weeks^2^	primary care	probability	occupation	↑	o
				specialist				o
	Spain			primary care				–
				specialist				+
Lu, et al. [42]	Hong Kong	≥ 16 years	1 months	primary care	probability	income	↑	+
					frequency			+
				specialist	probability			o
					frequency			o
Masseria, Giannoni [43]	Italy	> 16 years	4 weeks	primary care	probability	education	↑	–
						adjusted household income		+
				specialist		education		+
						adjusted household income		o
McDonald, Conde [44]	Canada	55 to 79 years	12 months	primary care	probability	education	↑	+
						adjusted household income		+
					conditional frequency	education		o
						adjusted household income		o
				specialist	probability	education		+
						adjusted household income		+
					conditional frequency	education		+
						adjusted household income		o
Mosquera, et al. [45]	Sweden	16 to 25 years	3 months	primary care	probability	household income	↑	–
Nolan [46]	Ireland	≥ 16 years	12 months	primary care	frequency	education	↑	o
						adjusted household income		o
Palència, et al. [47]	Spain	≥ 16 years	4 weeks	primary care	probability	occupation	↑	–
				specialist				+
Põlluste, Kalda, Lember [48]	Estonia	65 to 74 years	12 months	primary care	probability	education	↑	o
						adjusted household income		o
				specialist		education		o
						adjusted household income		o
Rattay et al. [49]	Germany	18 to 79 years	12 months	primary care	probability	index	↓	–
Regidor, et al. [50]	Spain	16 to 74 years	2 weeks	primary care	probability	education	↑	–
						adjusted household income		–
						occupation		–
				specialist		education		+
						adjusted household income		+
						occupation		+
Reibling, Wendt [51]	Austria, Belgium, Denmark, France, Germany, Greece, Italy, Netherlands, Spain, Sweden, Switzerland	≥ 50 years	12 months	specialist	probability	education	↑	+ ^5^
Rogowski et al. [52]	USA	≥ 65 years	12 months	primary care	frequency	education	↑	o
						adjusted household income		o
				specialist		education		+
						adjusted household income		o
Ryvicker, Gallo, Fahs [53]	USA	60 to 99 years	12 months	primary care	probability	education	↑	+
San Sebastian, Mosquera, Gustafsson [54]	Sweden	18 to 84 years	3 months	primary care	probability	income	↑	+
				specialist				o
Schnitzer, et al. [55]	Germany	18 to 79 years	12 months	specialist	frequency	education	↑	+
Schulz [56]	Pooled Data from 13 European Countries	≥ 40 years	12 months	primary care	frequency	education	↑	–
				specialist				+
Stirbu, et al. [11]	Belgium, Estonia, France, Germany, Hungary, Ireland, Latvia, Netherlands, Norway	≥ 15 years	12 months^3^	primary care	probability	education	↑	o^5^
				specialist				+
Suominen-Taipale, et al. [57]	Finland	65 to 74 years	12 months	primary care	probability	education	↑	o
				specialist				+
	Norway			primary care				+
				specialist				+
Tavares, Zantomio [58]	Italy	≥ 50 years	12 months	primary care	frequency	education	↑	–
				specialist				+
	Spain			primary care				–
				specialist				+
	Portugal			primary care				+
				specialist				+
Terraneo [10]	Pooled Data from 12 European Countries	≥ 50 years	12 months	primary care	probability	education	↑	o
				specialist				+
Thode et al. [59]	Germany	18 to 79 years	12 months	primary care	frequency	index	↑	–
Tille, et al. [60]	Germany	≥ 18 years	12 months	primary care	frequency	education	↑	–
				specialist				o
van Doorslaer, Koolman, Jones [61]	Austria, Belgium, Denmark, Germany, Greece, Ireland, Italy, Luxemburg, Netherlands, Portugal, Spain, UK	≥ 16 years	12 months	primary care	probability	adjusted household income	↑	o^5^
					frequency			−^5^
					conditional frequency			−^5^
				specialist	probability			+ ^5^
					frequency			+ ^5^
					conditional frequency			o^5^
van Doorslaer, Masseria, Koolman [62]	Austria, Belgium, Canada, Denmark, Finland, France, Germany, Greece, Hungary, Ireland, Italy, Netherlands, Norway, Portugal, Spain, Switzerland, UK	≥ 16 years	12 months	primary care	probability	adjusted household income	↑	o^5^
					conditional frequency			−^5^
				specialist	probability			+
					conditional frequency			+ ^5^
van Ourti [63]	Belgium	> 15 years	12 months	primary care	frequency	adjusted household income	↑	–
				specialist				o
Vasquez, Paraje, Estay [64]	Chile	≥ 18 years	3 months	primary care	probability	adjusted household income	↑	+
					frequency			+
				specialist	probability			+
					frequency			+
Vedsted et al. [65]	Denmark	20 to 34 years	12 months	primary care	conditional frequency	education	↑	o
		35 to 49 years						o
		50 to 64 years						o^4^
Vedsted, Olesen [66]	Denmark	20 to 64 years	12 months	primary care	conditional frequency	education	↑	o^4^
Vikum, et al. [67]	Norway	≥ 20 years	12 months	primary care	probability	education	↑	o^4^
		20 to 67 years				income		o^4^
		≥ 20 years		specialist		education		+
		20 to 67 years				income		+
Vikum, Krokstad, Westin [68]	Norway	≥ 20 years	12 months	primary care	probability	education	↑	–
						adjusted household income		o
				specialist		education		+
						adjusted household income		+

^1^, deviating for probability in Denmark: 3 months; and for frequency in some EHIS countries: 4 weeks

^2^, deviating for outpatient consultations in UK: 3 months

^3^, deviating for consultations in Netherlands and Belgium: 2 months

^4^, significant results only in gender-specific subgroup-analyses

^5^, results differ for several countries, only the most frequent results are reported

^6^, only for private specialists; results for hospital outpatient visits are non-significant

+, higher utilization in the most advantaged; o, no significant differences; -, higher utilization in the most deprived

↑, adjustment for at least gender, age and any general health variable (e.g., self-rated health or chronic conditions); ↓, relevant adjustments for need have not been conducted

### Socioeconomic differences in primary-care and specialist physician visits

**Fig. 2:**
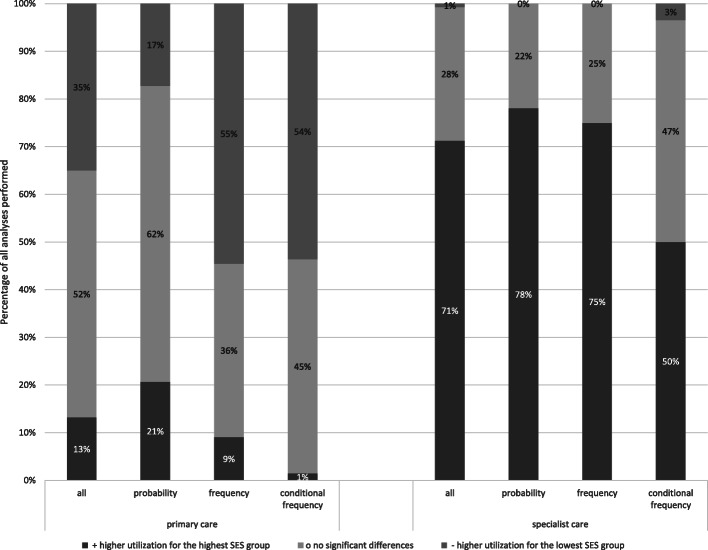
Socioeconomic differences in primary-care and specialist physician visits in all analyses, and subdivided for the different operationalisations of utilization (probability, frequency, and conditional frequency)

Overall, 52% of the analyses on utilization of primary-care physicians found no inequalities, and 35% found higher utilization for the lowest SES group (Fig. 2; primary care ‘all’). Contrary, 71% of the analyses on utilization of specialist physicians found higher utilization for the highest SES group, and 28% found no inequalities (Fig. 2; specialist care ‘all’). While taking a closer look at the various measures of utilization (Fig. 2; probability, frequency and conditional frequency), we found that 62% of the analyses on the probabilities of utilizing a primary-care physician found no socioeconomic inequalities, while 55% of the analyses on frequencies, and 54% of the analyses on conditional frequencies of primary-care physician visits found higher utilization in the most deprived. The results on specialist physicians also differed according to the operationalization of utilization in the way that 78% of the analyses on the probability and 75% of the analyses on the frequency of specialist visits found higher utilization for the highest SES-group. Higher utilization for the highest SES-group was found in only 50% of the analyses on the conditional frequency of specialist visits, whereas another 47% of the latter found no inequalities.

### Various measures of socioeconomic differences in physician utilization

In a second step, we took a closer look at further variations of measures, in order to examine whether they might cause distinct results. Therefore, we contrasted the study’s results (additional file 2) according to differences in time periods for which physician utilization was reported, and SES indicators.

Regarding time periods of utilization, it was found that in shorter time periods of 6 months or less, higher probabilities for primary-care physician visits in the lowest SES-group emerged, whereas studies analysing longer time periods found more often no inequalities. Contrariwise, for specialist visits higher probabilities in the highest SES-group were found less often in short compared to long time periods. Because only a very limited number of studies analysed the frequency or conditional frequency of utilization in a short time period, we renounced the comparison.

Regarding different SES indicators, higher probabilities and frequencies of primary-care physician visits were found for those with low education compared to those with low income, but higher conditional frequencies of primary-care physician visits were found more often for those with low income compared to those with low education. Results for socioeconomic inequalities in specialist physician visits seemed to hardly differ according to SES measurement. As only few studies measured SES by occupation or by an index, we renounced the comparison.

### Quality of the studies

The quality of the included studies was fairly high, as the majority of the studies was rated to have a low risk of bias in at least five of the six domains of RoBANS. Only three studies were ranked to have a high risk of bias in more than one domain (additional file 3). The risk of bias of *confounding variables, measurement of exposure*, *blinding of outcome measure* and *selective outcome reporting* was rated low in 54 studies, whereas the risk of bias of *incomplete outcome data* was rated high in 23 studies (additional file 3).

## Discussion

### Principal findings

In general, socioeconomic inequalities in utilization of physicians were more prevalent among specialists than among primary-care physicians. The probability of utilizing primary care was often not influenced by SES in the general population, but the disadvantaged visited their primary-care physician more frequently. Moreover, the highest-SES groups often had higher probabilities for specialist visits, but studies often found no associations of SES with (conditional) frequencies of specialist visits.

### Interpretation

This systematic review confirms that the existence of socioeconomic differences in healthcare utilization heavily depends on the health services analysed [9]. The existing review on socioeconomic inequalities in physician visits in the elderly population, which did not differentiate between primary-care and specialist physicians, found more medical appointments for the highest-SES group [9]. Accordingly, we found that a distinction of medical appointments between primary and specialized care is necessary when analysing socioeconomic inequalities in physician visits, because the results differed greatly according to the type of doctor and the type of service. We found that not all medical appointments, but mainly specialist were visited with higher probabilities and frequencies by the highest-SES groups. In contrast, most studies indicated that the probability of visiting primary-care physicians was not determined by SES, comparable to the evidence for hospitalization and emergency use, which rather presents access to need- and emergency-oriented healthcare [9]. Lastly, the frequency of primary-care physician visits often was higher in the lowest-SES groups, and is therefore comparable to the evidence on inequalities in utilization of home health services und visits [8, 9].

Consequently, socioeconomic inequalities disadvantaging the deprived are a matter of concern especially in specialist visits. Based on this review, we are not able to infer whether these inequalities are a matter of need, a matter of access barriers to specialist physicians, a matter of different information, or a matter of different preferences and patient choice. Nevertheless, nearly all studies adjusted for patient’s need according to gender, age, and any physical health condition. Either self-rated health or the number of self-reported chronic conditions was applied as an indicator for the latter. Although this indicates good quality, these indicators remain only approximate to real need of receiving healthcare. Accordingly, we cannot conclude that probability of primary-care physician visits is needs-based even though most studies did not find significant associations with SES. In order to avoid underestimating or disregarding differences, when analysing only probabilities of visits using register-based data, Agerholm et al. concluded that health status should be considered in analyses on socioeconomic differences in healthcare utilization [16]. Nevertheless, self-rated health remains a subjective rating of people’s perception of their health. Although studies found that self-rated health is a good proxy for objective health in the general population, [69] one study found that the evaluation of self-rated health is biased by SES, because the more educated rated their subjective health worse with the same level of objective health, [70] which implies socioeconomic differences might be underestimated.

Given the results that low-SES populations often visit specialist physicians less often, but primary-care physicians more frequently at concurrently equal probabilities compared to high SES populations, an intuitive explanation is that barriers in access to specialists are important in explaining healthcare inequalities. One possible reason for access barriers to specialists might be rurality of low-SES populations [71]. Thus, waiting time and distance might carry more weight in visiting specialists, because those are often distributed regionally more widely than primary-care physicians. Furthermore, the results suggest that different information, preferences, and patient choices are relevant reasons for socioeconomic inequalities in physician visits, because the relationship with primary-care physicians is more trusting and familiar than with specialist. As a consequence, the lower educated might feel less exposed to existing communication problems (language barriers, terminology, information gap) [72] with their longtime, well-known primary-care physicians, and they might prefer visits to them compared to specialist physicians [10]. The perceived role in healthcare varies between SES groups, as those with low SES tend to delegate responsibility to healthcare professionals [73]. Given the trustful and longtime relationship with primary care physicians compared to specialists, and given that primary-care physicians have the task of gatekeeping in some countries, might emphasize the importance of primary-care physicians from the perspective of the low-SES population when delegating responsibility for their healthcare, and might therefore explain the more frequent visits from the most deprived.

This review found that income inequalities advantaging high-SES groups in primary-care physician visits are more pronounced than educational differences. This may be an indication that financial barriers are a relevant additional factor explaining socioeconomic inequalities in utilization of primary-care physicians [74]. The finding that detrimental inequalities were found less often in shorter time periods is consistent with the finding that detrimental inequalities were found less often when utilization was operationalized with frequency versus probability. Accordingly, a higher frequency of physician visits among the most deprived means that they are more likely to have visited a physician at least once in a short period. A possible explanation could be that frequencies are more likely to be influenced by preferences and patient choice, whereas probabilities are more likely to be influenced by access barriers.

### Limitations

Although we have screened 1229 references we might have missed relevant publications, especially those not differentiating between primary and specialized healthcare in the abstract, but only in the main text. Second, the selection criteria might bias the results, which are not generalizable to children, disease-specific populations, low- and middle-income-countries, or inequalities induced by other (horizontal) disadvantages. Because very few studies based SES on area data, we excluded them even when area SES was linked to the individual on postal codes. Third, we made various simplifications in order to compare the studies, which influenced the reported results, which must be interpreted carefully. We described the results only by comparing the highest with the lowest SES group, and we did not include effect sizes in our descriptions. The health systems of the countries are very different, e.g., primary und specialized healthcare was defined differently in different studies. Primary health and its connection with specialist care is organised differently between the countries. For these reasons and because some studies analysed the same data basis, frequencies must be interpreted with caution, and comparisons are rather explorative hints than robust results. Finally, data on utilization, SES, and health were often self-rated, and even though instruments are valid, the accuracy is affected by different factors, [75] which limits expressiveness.

## Conclusions

In order to tackle socioeconomic inequalities in healthcare to the detriment of the deprived population, utilization of and access to specialist physicians is essential. The fact that predominantly no inequalities in probabilities of visiting primary-care physicians were found is generally a good result. Not visiting a primary-care physician can be interpreted as more fatal in maintaining good health than visiting specialists less frequently. This emphasizes the fact that the general population in high-income countries might have access to physicians largely independent of their SES, but the deprived might experience more barriers in accessing specialized healthcare. We assume that higher frequencies of primary-care physician visits from the low-SES groups with the same level of need might be subject to patient preferences in order to compensate for different levels of health literacy, information and communication, and therefore improve equal opportunities in receiving health maintenance.

## Supplementary Information

**Additional file 1.** Search Strategy. Search strategy used to identify articles in the Medline and Web of Science database including search terms, search strings, and filters.

**Additional file 2.** Figures of additional results. Figures of the results on socioeconomic differences in the probabilities of utilizing primary-care and specialist physicians (a) subdivided for the different time periods of utilization; (b) subdivided for the different measures of socioeconomic status.

**Additional file 3.** Results of the risk of Bias evaluation for (a) each article included; (b) each of each of the six domains of the risk of bias assessment.

## Data Availability

Not applicable.

## Abbreviation

SES: Socioeconomic Status
