# Supplementary material for: Socioeconomic inequalities in primary-care and specialist physician visits: a systematic review
Source: Int J Equity Health. 2021 Feb 10;20:58. doi: 10.1186/s12939-020-01375-1 (PMC7874661; doi:10.1186/s12939-020-01375-1)
Supplement: Supplementary file 1 — Additional file 1. Search Strategy. Search strategy used to identify articles in the Medline and Web of Science database including search terms, search strings, and filters. [file 12939_2020_1375_MOESM1_ESM.pdf]

Additional file 1 Search strategy

Medline
Search ((((((((((inequit*[Title/Abstract]) OR inequal*[Title/Abstract]) OR differences[Title/Abstract]) OR disparit*[Title/Abstract]) OR equit*[Title/Abstract]) OR equalit*[Title/Abstract])))))) AND ((((((((((((((socioeconomic[Title/Abstract]) OR socio-economic[Title/Abstract]) OR education*[Title/Abstract]) OR income[Title/Abstract]) OR "social class"[Title/Abstract]) OR occupation*[Title/Abstract] OR depriv*[Title/Abstract]) OR area[Title/Abstract]) OR poverty[Title/Abstract]))))))))) AND ((((((((((("General practitioner*"[Title/Abstract]) OR "family doctor*"[Title/Abstract]) OR "primary care physician*"[Title/Abstract]) OR "family physician*"[Title/Abstract]) OR "GP*"[Title/Abstract]) OR "primary physician*"[Title/Abstract]) OR "family practitioner*"[Title/Abstract]) OR "attending physician*"[Title/Abstract]) OR "medical specialist*"[Title/Abstract]) OR "consultant*"[Title/Abstract]) OR "specialist physician*"[Title/Abstract])) Filters: Publication date from 2004/01/01 to 2018/12/31; English; German

Web of Science
1 TI=(inequit*) OR TI=(inequal*) OR TI=(differences) OR TI=(disparit*) OR TI=(equit*) OR TI=(equalit*)
2 TI=(socioeconomic) OR TI=(socio-economic) OR TI=("social class") OR TI=(occupation*) OR TI=(income) OR TI=(education*) OR TI=(poverty) OR TI=(depriv*) OR TI=(area)
3 TI=("General practitioner*") OR TI=("family doctor*") OR TI=("primary care physician*") OR TI=("family physician*") OR TI=(GP) OR TI=("primary physician*") OR TI=("family practitioner*") OR TI=("attending physician*") OR TI=("medical specialist*") OR TI=(consultant*) OR TI=("specialist physician*")
#3 AND #2 AND #1
Refined by: LANGUAGES: (ENGLISH OR GERMAN)
Timespan=2004-2018
Search language=Auto
