# Supplementary material for: Socioeconomic inequalities in primary-care and specialist physician visits: a systematic review
Source: Int J Equity Health. 2021 Feb 10;20:58. doi: 10.1186/s12939-020-01375-1 (PMC7874661; doi:10.1186/s12939-020-01375-1)

Additional file 2a Socioeconomic differences in the probabilities of utilizing primary-care and specialist physicians subdivided for different time periods of utilization (≤6 months compared to 12-24 months)

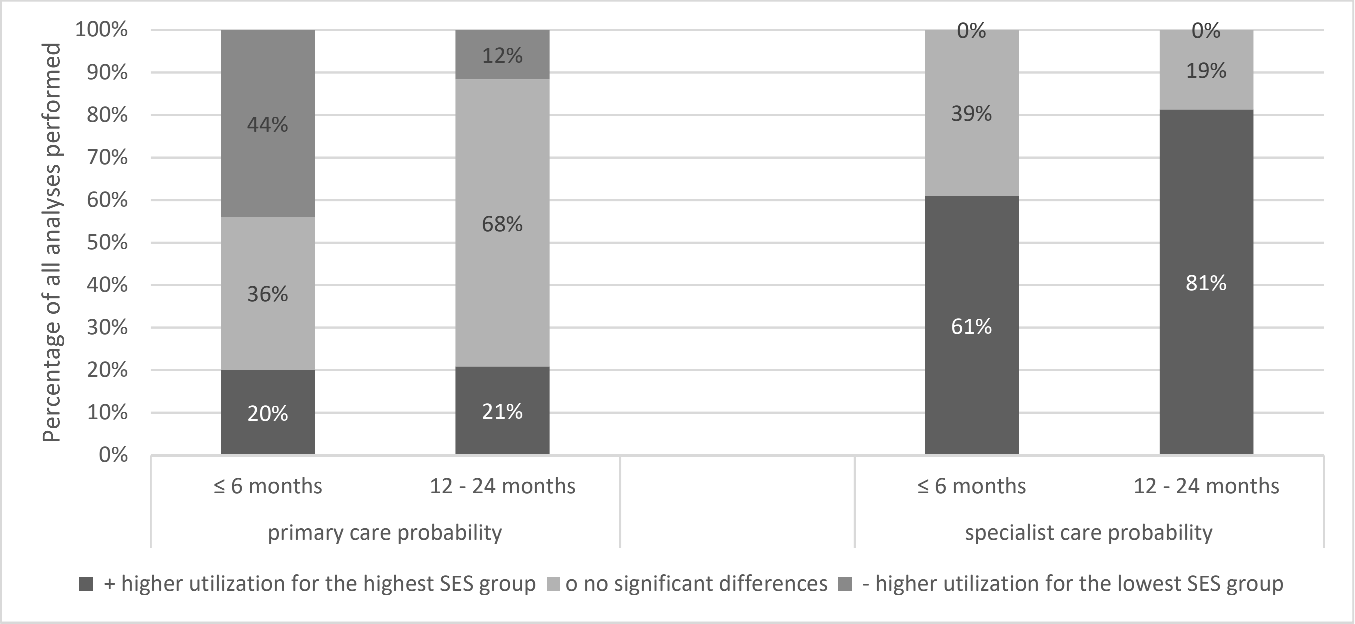

Additional file 2b Socioeconomic differences in the utilization of primary-care and specialist physicians subdivided for the different measures of socioeconomic status (income compared to education)

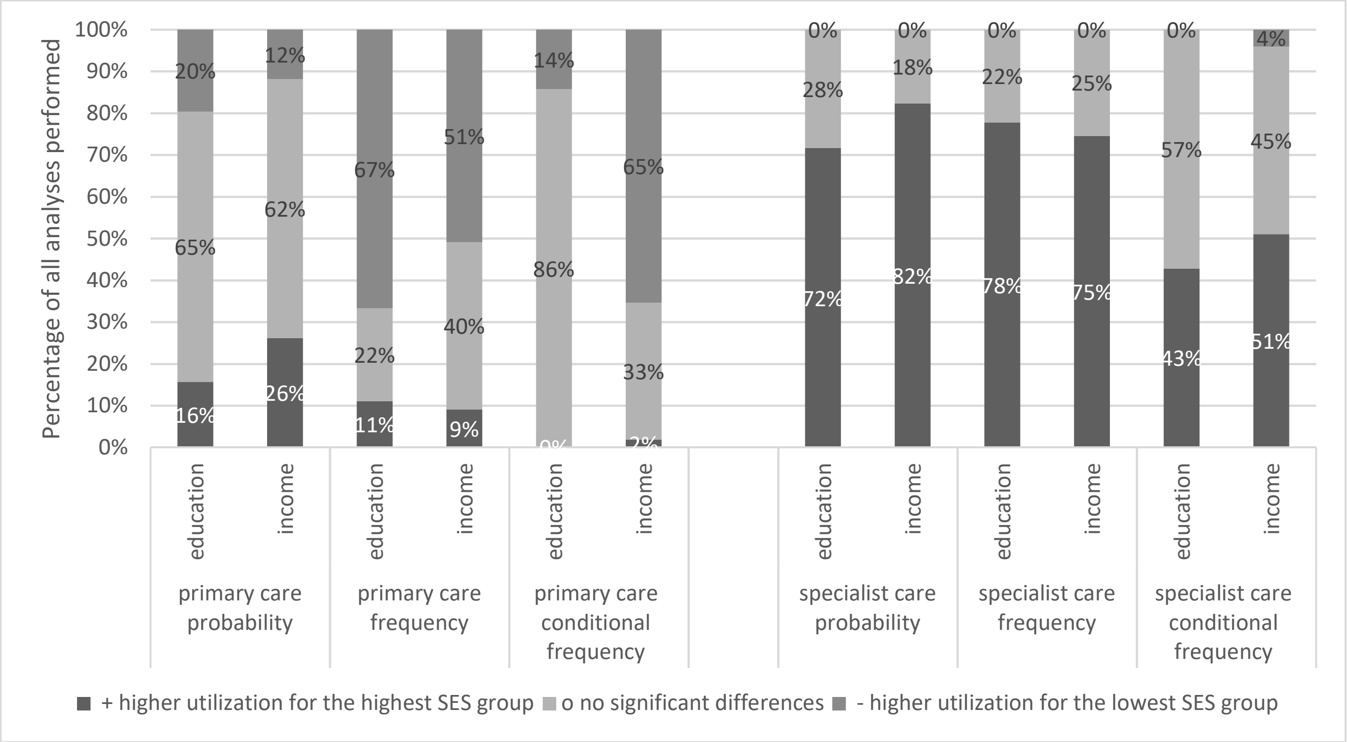

Supplement: Supplementary file 2 — Additional file 2. Figures of additional results. Figures of the results on socioeconomic differences in the probabilities of utilizing primary-care and specialist physicians (a) subdivided for the different time periods of utilization; (b) subdivided for the different measures of socioeconomic status. [file 12939_2020_1375_MOESM2_ESM.pdf]
