# Supplementary material for: Socioeconomic inequalities in primary-care and specialist physician visits: a systematic review
Source: Int J Equity Health. 2021 Feb 10;20:58. doi: 10.1186/s12939-020-01375-1 (PMC7874661; doi:10.1186/s12939-020-01375-1)
Supplement: Supplementary file 3 — Additional file 3. Results of the risk of Bias evaluation for (a) each article included; (b) each of each of the six domains of the risk of bias assessment. [file 12939_2020_1375_MOESM3_ESM.pdf]

Additional file 3a Results of the risk of Bias evaluation

Author	Year	#1	#2	#3	#4	#5	#6
Abasolo, Saez, López-Casasnovas[23]	2017	Green	Red	Green	Green	Red	Red
Agerholm et al.[15]	2013	White	Green	Green	Green	Red	Green
Allin[24]	2008	Green	Green	Green	Green	Green	Green
Asada, Kephart[25]	2007	White	Green	Green	Green	Green	Green
Bago d'Uva, Jones, van Doorslaer[26]	2009	Green	Green	Green	Green	White	Green
Baron-Epel, Garty, Green[27]	2007	Green	Green	Green	Green	Red	Green
Beckman, Anell[28]	2013	Green	Red	Green	Green	Green	Green
Bergmann, Kalcklösch, Tiemann[29]	2005	Red	Green	Green	Green	Green	Green
Bourke[30]	2009	Green	Green	Green	Green	White	Green
Bremer, Wübker[31]	2013	Green	Green	Green	Green	White	Green
Bremer, et al.[32]	2018	Green	Green	Green	Green	Red	Green
Crespo-Cebada, Urbanos-Garrido[33]	2012	Green	Green	Green	Green	White	Green
Devaux, de Looper[34]	2012	Green	Green	Green	Green	White	Green
Fjaer, et al.[35]	2017	White	Green	Green	Green	Red	Green
Garrido-Cumbrera, et al.[36]	2010	Green	Green	Green	Green	White	Green
Glazier et al[37]	2009	White	Green	Green	Green	Red	Green
Gonzalez-Alvarez, Barranquero[38]	2009	Green	Green	Green	Green	White	Green
Grasdal, Monstad[39]	2011	Green	Green	Green	Green	Red	Green
Gruber, Kiesel[40]	2010	Green	Green	Green	Green	Red	Green
Habicht, Kunst[41]	2005	White	Green	Green	Green	Red	Green
Hansen, et al.[42]	2012	Green	Green	Green	Green	White	Green
Hoebel, et al.[12]	2016	Green	Green	Green	Green	Red	Green
Hoeck, et al.[43]	2011	Green	Green	Green	Green	White	Green
Hoeck, et al.[44]	2013	Green	Green	Green	Green	Green	Green
Korda, et al.[45]	2009	White	Green	Green	Green	Green	Green
La Parra-Casado, et al.[46]	2018	Green	Green	Green	Green	White	Green
Lichte[47]	2017	Green	Green	Green	Green	White	Green
Lostao, et al.[48]	2011	Green	Green	Green	Green	White	Green
Lu, et al.[49]	2007	Green	Green	Green	Green	White	Green
Masseria, Giannoni[50]	2010	Green	Green	Red	Green	White	Green
McDonald, Conde[51]	2010	White	Green	Green	Green	White	Green
Mosquera, et al.[52]	2017	Green	Green	Green	Green	White	Green
Nolan[53]	2007	Green	Green	Green	Green	Red	Green
Palència, et al.[54]	2013	Green	Green	Green	Green	Red	Green
Põlluste, Kalda, Lember[55]	2009	Green	Green	Green	Green	Red	Green
Rattay et. al.[56]	2013	Green	Red	Green	Green	Red	Green
Regidor, et al.[57]	2008	White	Green	White	Green	White	Green
Reibling, Wendt[58]	2010	Green	Green	Green	Green	White	Green
Rogowski et al.[59]	2008	Green	Green	Green	Green	Green	Green
Ryvicker, Gallo, Fahs[60]	2012	Green	Green	Green	Green	Red	Green
San Sebastian, Mosquera, Gustafsson[61]	2017	White	Green	Green	Green	White	Green
Schnitzer, et al.[62]	2011	Red	Green	Green	Green	Red	Green
Schulz[63]	2016	Green	Green	Green	Green	White	Green
Stirbu, et al.[11]	2011	Green	Green	Green	Green	White	Green
Suominen-Taipale, et al.[64]	2004	Green	Green	Green	Green	Red	Green
Tavares, Zantomio[65]	2017	Green	Green	Green	Green	White	Green
Terraneo[10]	2015	Green	Green	Green	Green	Red	Green
Thode et al.[66]	2005	Green	Green	Green	Green	White	Green
Tille, et al.[67]	2017	Green	Green	Green	Green	Red	Green
van Doorslaer, Koolman, Jones[68]	2004	Green	Green	Green	Green	White	Green
van Doorslaer, Masseria, Koolman[69]	2006	Green	Green	Green	Green	White	Green
van Ourti[70]	2004	Green	Green	Green	Green	Red	Green
Vasquez, Paraje, Estay[71]	2013	Green	Green	Green	Green	White	Green
Vedsted et al.[72]	2004	White	Green	Green	Green	White	Green
Vedsted, Olesen[73]	2005	White	Green	Green	Green	Red	Green
Vikum, et al.[74]	2013	Green	Green	Green	Green	Red	Green
Vikum, Krokstad, Westin[75]	2012	Green	Green	Green	Green	Red	Green

#1 selection of participants; *#2 confounding variables*; *#3 measurement of exposure*; *#4 blinding of outcome measure*. *#5 incomplete outcome data*; *#6 selective outcome reporting*.
Green, low risk of bias; white, unclear risk of bias; red, high risk of bias

Additional file 3b Evaluation of each of the six domains of the risk of bias assessment

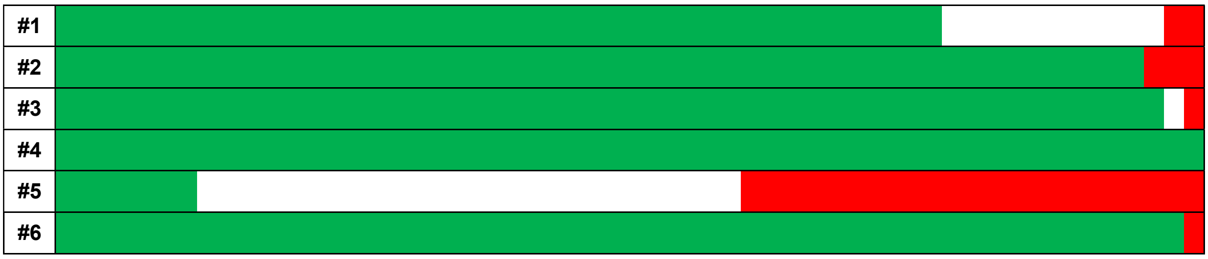

#1 selection of participants; #2 confounding variables; #3 measurement of exposure; #4 blinding of outcome measure. #5 incomplete outcome data; #6 selective outcome reporting.

Green, low risk of bias; white, unclear risk of bias; red, high risk of bias
